# Naphthalimide Derivatives with Extended Heterocyclic Systems—Synthesis, Spectral and Sensing Properties

**DOI:** 10.3390/s26072236

**Published:** 2026-04-04

**Authors:** Hristo Manov, Ivo Grabchev, Yulian Zagranyarski, Diana Cheshmedzhieva, Ivan Atanasov, Monika Mutovska, Konstantin Konstantinov, Stanimir Stoyanov

**Affiliations:** 1 Faculty of Chemistry and Pharmacy, Sofia University “St. Kliment Ohridski”, 1 J. Baurchier Blvd., 1164 Sofia, Bulgaria; xgmanov@gmail.com (H.M.); ohjz@chem.uni-sofia.bg (Y.Z.); dvalentinova@chem.uni-sofia.bg (D.C.); i.atanas.031@gmail.com (I.A.); ohmgm@chem.uni-sofia.bg (M.M.); 2 Faculty of Medicine, Sofia University “St. Kliment Ohridski”, 1 Koziak Str., 1407 Sofia, Bulgaria; 3 Faculty of Pharmacy, Medical University of Sofia, 2 Dunav Str., 1000 Sofia, Bulgaria; k.konstantinov@pharmfac.mu-sofia.bg

**Keywords:** 1,8-naphthalimides, heterocyclic annulation, photoinduced electron transfer, fluorescent sensors, metal ion sensing, density functional theory

## Abstract

**Highlights:**

**What are the main findings?**

- New π-extended naphthalimide fluorophores were synthesized and evaluated as PET optical sensors for protons and metal cations.
- Strong fluorescence enhancement was observed for Cu(II) and Sn(II) due to efficient PET suppression confirmed by Job’s plot and DFT studies.

**What are the implications of the main findings?**

- Proper donor–acceptor orbital alignment is essential for achieving efficient switch-ON PET sensing.
- Heterocyclic annulation provides a useful strategy for tuning photophysical properties and selectivity of fluorescent probes.

**Abstract:**

The objective of this study was to design and evaluate π-extended 1,8-naphthalimide derivatives as photoinduced electron transfer (PET) optical sensors for protons and metal cations, with emphasis on the role of heterocyclic annulation and receptor–chromophore electronic matching. Benzofuran- and benzodioxin-annulated naphthalimides bearing either a dimethylaminoethyl receptor or a non-donating alkyl substituent at the imide nitrogen were synthesized using tailored synthetic strategies. Their photophysical properties were investigated by absorption and fluorescence spectroscopy, while sensing performance was evaluated by fluorescence titrations. Quantum chemistry calculations were employed to rationalize experimental observations. Benzofuran-annulated derivatives exhibit structured π–π* absorption bands and strong fluorescence, whereas introduction of the receptor induces efficient fluorescence quenching via reductive PET. Protonation or metal ion coordination suppresses PET and leads to pronounced fluorescence enhancement, particularly in the presence of Cu(II) and Sn(II). In contrast, benzodioxin-annulated derivatives display intramolecular charge-transfer absorption bands, large Stokes shifts, and low fluorescence quantum yields in polar media, resulting in a negligible sensing response. Computational results attribute this behavior to an unfavorable energy arrangement of the donor–acceptor orbitals. Overall, the study demonstrates that heterocyclic annulation critically governs the electronic structure and sensing performance of naphthalimide fluorophores, providing guidelines for the rational design of PET-based optical sensors.

## 1. Introduction

Fluorescent molecular sensors based on photoinduced electron transfer (PET) are widely used for the detection of protons, metal ions, and biologically relevant analytes due to their high sensitivity, fast response, and structural tunability. In PET-based probes, fluorescence modulation is achieved through controlled electronic interaction between a fluorophore and an electron-donating receptor, where analyte binding suppresses or activates the electron transfer process, resulting in a measurable change in emission intensity. Such systems are particularly attractive for the development of optical sensors operating in solution, in which selectivity and signal amplification are critical parameters [1,2,3].

Among the various fluorophores employed in PET sensors, 1,8-naphthalimide (NI) derivatives represent one of the most versatile platforms owing to their high photostability, favorable absorption and emission characteristics, and ease of structural modification [4,5]. Substitution at the imide nitrogen allows straightforward introduction of receptor fragments, while modification of the aromatic core enables fine tuning of the electronic structure and photophysical properties [6,7,8]. As a result, naphthalimide-based probes have been extensively investigated for sensing of metal cations, pH changes, and reactive species, as well as for biological imaging applications [9,10,11,12,13,14].

An efficient strategy for controlling the optical and sensing properties of naphthalimides is extension of the π-conjugated system through annulation of heterocyclic rings [15]. Such structural modification can significantly alter the electronic coupling within the chromophore, leading to changes in absorption wavelength, emission efficiency, and the balance between locally excited and charge-transfer states [16]. In particular, annulation with oxygen-containing heterocycles has been shown to produce fluorophores with markedly different spectral behavior, which may strongly influence the efficiency of PET processes and, consequently, the sensing performance [17,18,19].

For PET-based sensors, an essential requirement is the proper energetic alignment between the donor orbital of the receptor and the acceptor orbital of the fluorophore [20,21,22]. Even small structural modifications can disrupt this balance and lead to loss of sensing response despite similar molecular frameworks. Therefore, systematic comparison of structurally related chromophores with different modes of π-extension represents a useful approach for establishing structure–property relationships and for developing design principles for efficient optical sensors [23,24,25].

In the present work, we investigate the influence of heterocyclic annulation on the photophysical properties and PET sensing behavior of 1,8-naphthalimide derivatives bearing a dimethylaminoethyl receptor fragment. Benzofuran- and benzodioxin-annulated NIs with and without receptor units were synthesized using tailored synthetic approaches that overcome the solubility and purification limitations of highly conjugated imides. Their absorption and fluorescence properties were studied in solution, and their sensing performance toward protons and selected metal cations was evaluated by fluorescence titrations. Density functional theory (DFT) and time-dependent DFT calculations were employed to rationalize the observed spectral features, the efficiency of the PET process, and the different sensing responses of the two annulated systems.

## 2. Results and Discussion

### 2.1. Synthesis

The primary objective of our synthetic efforts was to obtain novel NI derivatives featuring an extended heterocyclic framework and to evaluate their applicability as PET optical sensors for protons and metal cations. Our studies in recent years have enabled the development of synthetic approaches for the annulation of furan and dioxin rings onto the naphthalimide core. For each class of derivatives, we aimed to prepare a pair of compounds: one incorporating a receptor fragment acting as an electron donor in the PET process and a corresponding analogue bearing a classical hydrocarbon substituent lacking such properties.

**Scheme 1:**
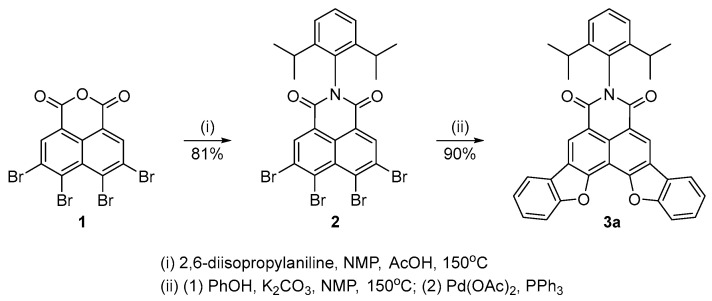
Synthesis of the reference difuranyl-annulated NI **3a**, as described in [27].

We recently demonstrated a method for the preparation of symmetrically benzofuran-annulated 1,8-naphthalimides from 3,4,5,6-tetrabromonaphthalic anhydride **1** via a one-pot Sn-Ar/C–H activation tandem reaction, providing access to new π-extended NI chromophores in only two steps and in high yields [26]. Using this streamlined approach, we successfully synthesized the *N*-(2,6-diisopropylphenyl) difuranyl NI derivative **3a** (Scheme 1).

**Scheme 2:**
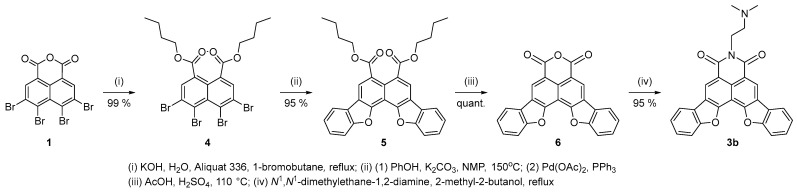
Synthesis of the target receptor-bearing difuranyl-NI **3b** from **6**. The first three steps are described in [27].

Imides bearing extended heterocyclic frameworks typically exhibit low solubility, necessitating the introduction of bulky substituents—such as the reported 2,6-diisopropylphenyl group or branched alkyl chains—to facilitate purification. All attempts to employ substituents of lower steric demand in precursor **2**, such as *n*-butyl or the target *N*,*N*-diaminoethyl, resulted in crude products that were impossible to purify by conventional methods. Therefore, we decided to apply the alternative synthetic route proposed in the same study, which relies on a diester intermediate (Scheme 2). This approach offers several key advantages, including a straightforward and efficient initial transformation, compatibility of the diester intermediate with the Sn-Ar/C–H activation tandem reaction in contrast to the corresponding anhydride, improved solubility of the difurano diesters enabling facile purification, and a final quantitative cyclization step that delivers a pure product **6** driven by anhydride precipitation. The latter was directly engaged in the imidization reaction with *N*^1^,*N*^1^-dimethylethane-1,2-diamine, affording the target compound **3b** in quantitative yield, thereby retrospectively confirming the high purity of the corresponding anhydride.

**Scheme 3:**
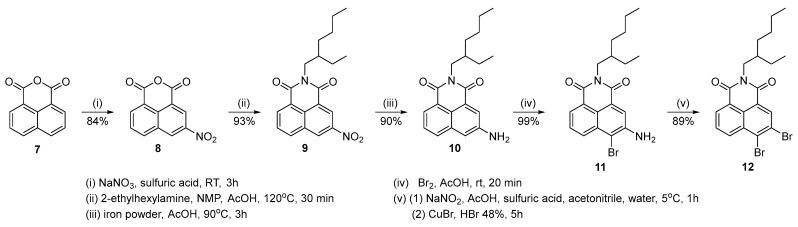
Synthesis of the building-block 3,4-dibromo NI **12**, as described in [29].

The annulation of a dioxin ring onto the NI core requires the use of different starting materials and reaction conditions, namely, a 3,4-dihalo-substituted NI and an ortho-dihydroxyarene, to enable a double nucleophilic substitution. This strategy was successfully demonstrated in our previous studies for the synthesis of new fluorophores for OLED applications [27]. Polyhalogenated naphthalimides, however, are not commercially available, and their preparation is often associated with significant challenges related to achieving the desired substitution pattern. Recently, we developed and reported an improved strategy for the gram-scale preparation of 3,4-dichloro- and 3,4-dibromo-NI, as well as mixed 3,4-dihalogenated bromo–chloro NIs, starting from inexpensive and commercially available 1,8-naphthalic anhydride [28]. Using this approach, 3,4-dibromo-*N*-(2-ethylhexyl) naphthalimide **12** was obtained at a very good overall yield and selected as the starting compound for the present derivatization (Scheme 3).

**Scheme 4:**
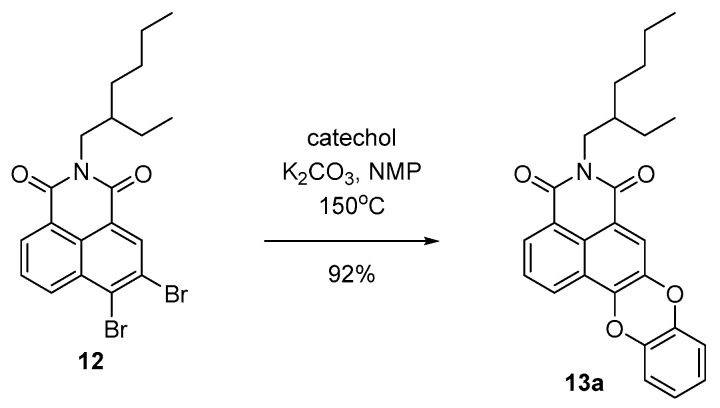
Synthesis of the reference benzodioxin-annulated NI **13a**.

With compound **12** in hand, we were able to perform the key dioxin-annulation step by subjecting it to a nucleophilic substitution reaction with catechol, conducted in NMP at 150 °C using potassium carbonate as the base under an inert atmosphere. Under these conditions, the reaction proceeded within only 50 min, as monitored by TLC. The target benzodioxine derivative **13a** was isolated and purified at a 92% yield (Scheme 4).

**Scheme 5:**
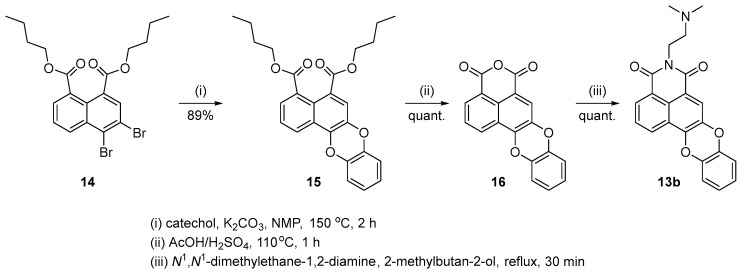
Synthesis of the target receptor-bearing benzodioxin-annulated NI **13b**.

With the reference benzodioxin-annulated naphthalimide **13a** in hand, the remaining task was the preparation of its analogue bearing an *N*,*N*-diaminoethyl receptor fragment. As in the benzofuran-annulated series, introduction of this functionality at an early synthetic stage resulted in substantial purification difficulties. Therefore, we again adopted a strategy based on diester intermediates for π-system extension, which were converted into the corresponding anhydrides and subjected to imidization only at the final stage (Scheme 5). This approach afforded the target compound **13b**, featuring a receptor unit with potential PET sensing properties, which was very recently reported as part of studies on the antitumor activity of a series of mitonafide and amonafide analogues [29].

### 2.2. Spectral Properties

**Figure 1:**
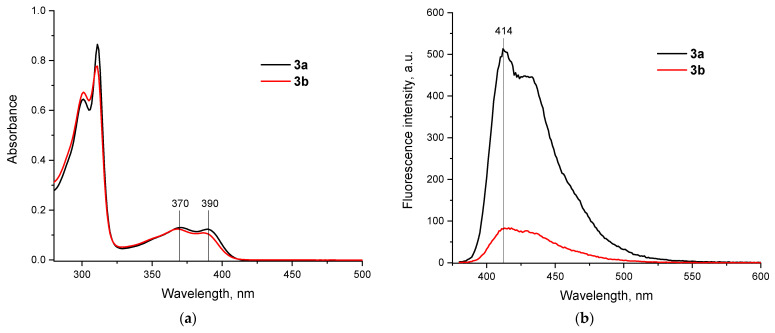
Absorption (**a**) and emission (**b**) spectra of **3a**,**b** in DMF, c = 1.10^−5^ M.

Both symmetrically benzofuran-annulated imides **3** exhibit a broad, structured absorption band in the 325–400 nm region, which can be assigned to a π–π* transition within the extended aromatic system. The corresponding absorption spectra are shown in Figure 1a. As can be seen, the nature of the substituent on the imide nitrogen has no influence on either the band position or intensity. The molar absorptivities exceed 10,000 L mol^−1^ cm^−1^, as expected for allowed transitions in such NI-based chromophores. Compound **3b** shows limited solubility in non-polar solvents; however, **3a**, examined in dichloromethane, did not exhibit any solvatochromic behavior. This observation further supports the assignment of the transition as being predominantly π–π* in character.

In contrast, the emission behavior of imides **3** differs markedly (Figure 1b). The reference compound **3a** emits blue fluorescence with an intense maximum at 414 nm and a quantum yield of 27%. The receptor-containing analogue **3b** displays an emission band of identical shape and position but with significantly reduced intensity, resulting in a quantum yield below 5%. The pronounced fluorescence suppression without any change in emission wavelength is a clear indication of an operative reductive PET process from the dimethylamino receptor to the excited NI chromophore, effectively quenching the fluorescence. The efficiency of the PET process can be assessed using the fluorescence quenching (FQ) factor, defined as the ratio of the respective quantum yields, which is relatively high for this compound pair (FQ = 6.1). The photophysical characteristics of all the investigated NIs are summarized in Table 1.

**Figure 2:**
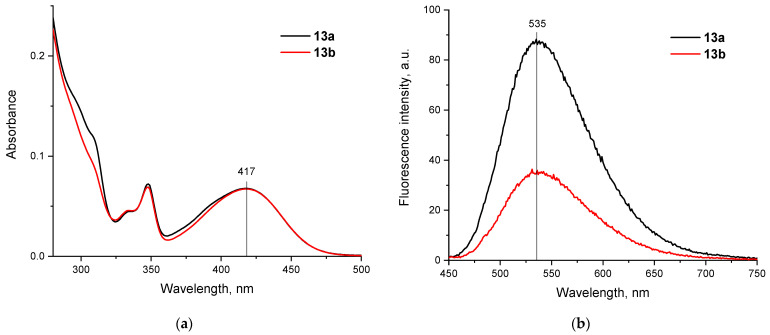
Absorption (**a**) and emission (**b**) spectra of **13a**,**b** in DMF, c = 1.10^−5^ M.

The substitution pattern in the second studied compound pair differs substantially, resulting in pronounced changes in their spectral properties. In this case, the benzodioxin moiety does not form a unified aromatic system with the NI core; instead, the two oxygen atoms can be regarded as auxochromic substituents attached to the naphthalimide scaffold, giving rise to a donor–π–acceptor-type system. This electronic arrangement is manifested by a broad, structureless absorption band in the blue region, with a maximum at 417 nm, which can be assigned to an intramolecular charge-transfer (ICT) transition (Figure 2a). As observed for the benzofuran-annulated derivatives, the nature of the substituent on the imide nitrogen has no influence on the absorption characteristics, which are virtually identical for **13a** and **13b**, displaying typical molar absorptivities of approximately 6700 L mol^−1^ cm^−1^.

The emission spectra of both dioxin-annulated NIs are identical in terms of band shape and position, exhibiting green fluorescence with an emission maximum at 535 nm (Figure 2b). However, a fluorescence suppression is observed for the receptor-bearing compound **13b**, consistent with the occurrence of reductive PET in the excited state, albeit with lower efficiency than in the other compound pair, as reflected by a fluorescence quenching factor of FQ = 2.5. Notably, the fluorescence quantum yield measured in DMF is low even for the reference compound **13a** (ca. 5%), which is characteristic of ICT systems in polar solvents. Such environments often favor the formation of non-emissive charge-separated or locally excited (LE) states, whereas in non-polar media, structurally related systems typically exhibit much higher fluorescence efficiencies [27]. This behavior is further manifested by the large Stokes shifts exceeding 5000 cm^−1^, indicating a substantial energy difference between the initially formed Franck–Condon excited state and the more strongly stabilized, polar LE state.

### 2.3. Molecular Modeling

**Figure 3:**
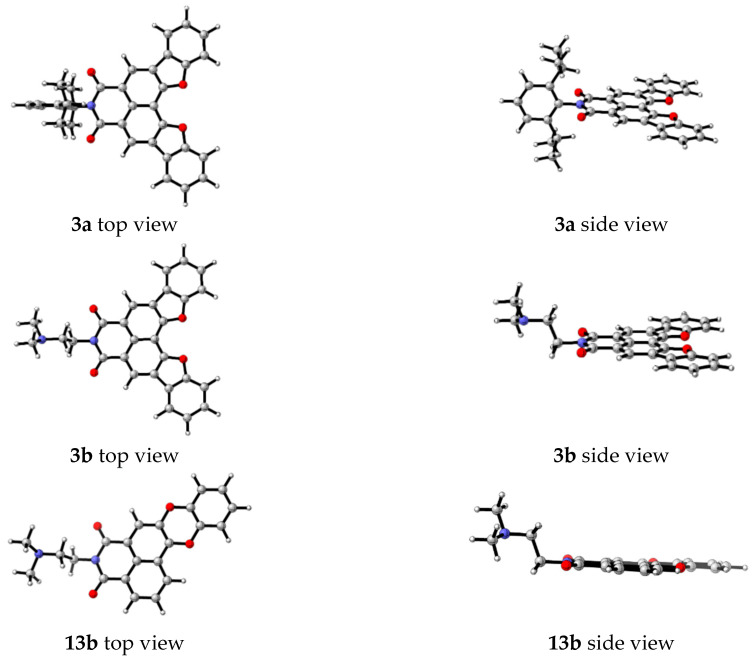
PBE0/6-311+G(2d,p)-optimized geometries of the studied compounds in DMF [33].

To further elucidate the nature of the electronic transitions and assess the influence of the substituents on the molecular geometry and spectral properties of the investigated compounds, DFT [30] and TDDFT [31] calculations were performed. The geometry optimization and photophysical properties of compounds were modeled with G16 software package [32]. The PBE0/6-311+G(2d,p)-optimized structures of **3a** and **3b** in DMF are shown in Figure 3.

The theoretical results for the absorption transition energies obtained with PBE0/6-311+G(2d,p) basis sets in DMF solution for the studied naphthalimides are collected in Table 2. A detailed description of the calculation procedure can be found elsewhere [34]. Solvent effects were examined at each step by means of PCM formalism [35]. Substitution at the imide nitrogen atom usually improves the solubility of the compounds, and modulates their aggregation properties in the solid state, but has a very weak effect on their spectral properties as seen from the experimental and theoretical data (Table 1 and Table 2). The combination of functional and basis sets used for the spectral calculations proved to be suitable for the compounds studied, as seen from the data in Table 2.

**Figure 4:**
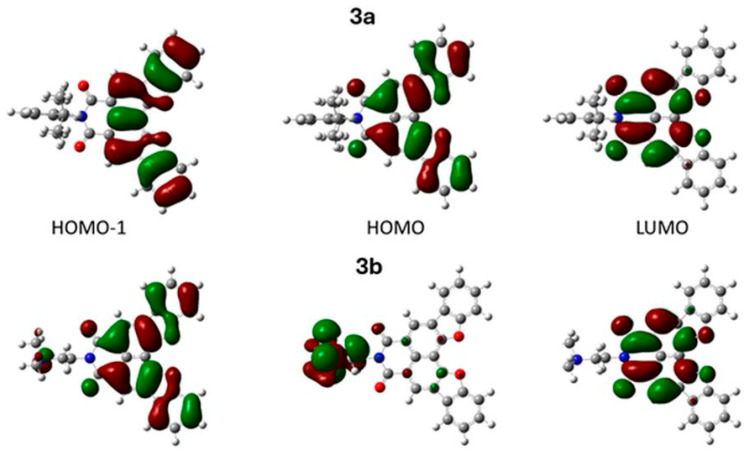
Shape representation of the ground state orbitals of **3a** and **3b** in DMF from TDPBE0/6-311+G(2d,p) computations—HOMO−1, HOMO, and LUMO.

**Figure 5:**
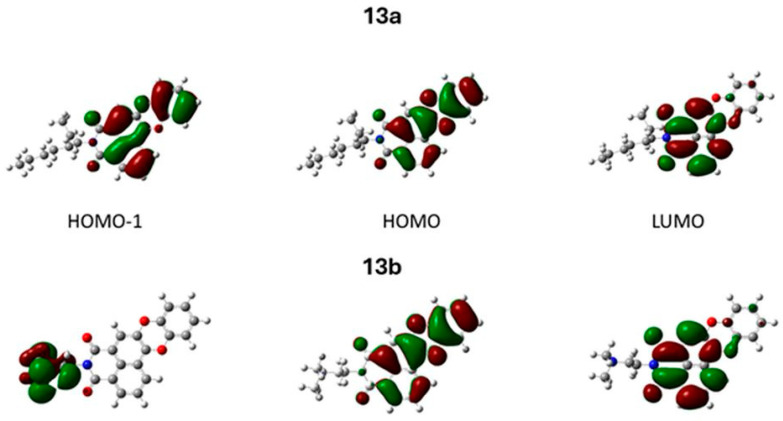
Shape representation of the ground state orbitals of **13a** and **13b** in DMF from TDPBE0/6-311+G(2d,p) computations—HOMO−1, HOMO, and LUMO.

The trends of changes in electron densities can be illustrated through an analysis of the molecular orbitals’ shape. The frontier molecular orbitals for the studied compounds in DMF are represented in Figure 4 and Figure 5.

The HOMO of **3a** is delocalized over the naphthalimide core and the oxygen-containing donor moiety, whereas the LUMO is more localized on the acceptor part of the molecule. The shape of the frontier orbitals indicates a short-range intramolecular charge transfer from the donor group toward the imide fragment (HOMO → LUMO transition). However, the dipole moment changes only negligibly upon excitation (μ = 6.1 D in the ground state, and μ = 6.2 D in the excited state), which explains the absence of solvatochromism for this chromophore.

As can be seen from Table 2 (second entry), in **3b** the transition with the highest oscillator strength that corresponds to the one in the experimental spectrum arises from HOMO−1 → LUMO. The electron density of HOMO is distributed over the N-substituents’ moiety—the receptor, whereas HOMO-1 is localized on the fluorophore (Figure 4). The lone pair orbitals of the amino groups in the receptor are of higher energies than those of the fluorophore, which is a precondition for a typical reductive PET process. The difference between the energy of orbitals of the receptor (HOMO) and the one of the chromophore (HOMO-1) is 0.3 eV, suggesting that, when excitation occurs, an electron would be transferred from the receptor to the fluorophore, which will lead to the quenching of **3b** emission.

The excited state geometries for **3a** and **3b** were optimized at the PBE0/6-311+G(2d,p) level. For **3a** the predicted fluorescence maxima in DMF is 2.98 eV (416 nm), which is in good agreement with the experimental value (414 nm, 2.99 eV). For **3b** the predicted fluorescence maxima in DMF is 3.13 eV (396 nm).

For compounds **13a** and **13b**, the electronic transition responsible for the experimental absorption band corresponds to a HOMO → LUMO excitation (Table 2). In these systems, the receptor orbital is located energetically below the chromophore HOMO (Figure 5), which prevents photoinduced electron transfer and explains the negligible PET effect in these compounds.

### 2.4. Optical Sensing

**Figure 6:**
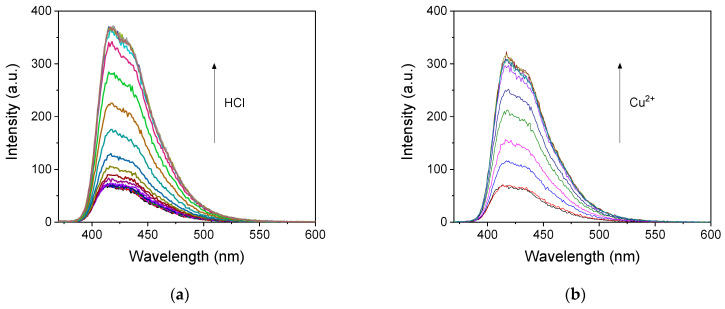
Fluorescence enhancement upon addition of acid (**a**) and Cu^2+^ (**b**) to a 1.10^−5^ M solution of **3b** in DMF.

The optical sensing capabilities of the receptor-bearing compounds **3b** and **13b** were evaluated by fluorescence titration experiments with acidic and metal salt solutions. According to the well-established operating mechanism of Switch-ON fluorescent PET sensors, engagement of the receptor lone electron pair upon analyte binding suppresses the PET process, thereby restoring fluorescence emission. Consequently, in the presence of protons or metal cations, the emission intensity increases (Figure 6).

**Figure 7:**
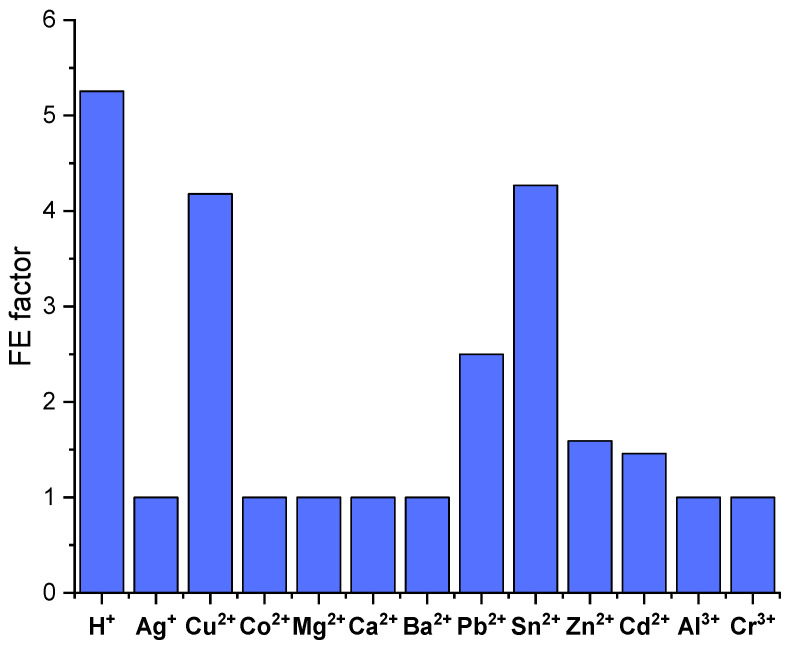
FE factors of **3b** upon addition of different analytes.

The sensor sensitivity was quantified using the fluorescence enhancement (FE) factor, defined as the ratio of the emission intensity at analyte saturation to the intrinsic fluorescence intensity of the sensor in the absence of analyte. As shown in Figure 7, the FE factors of **3b** strongly depend on the nature of the analyte. Addition of HCl results in more than a fivefold increase in emission intensity, rendering it comparable to that of the reference compound **3a**, as protonation by a strong acid suppresses the PET process to the maximum extent. Furthermore, fluorescence measurements over a broad pH range indicate that the sensor remains stable, with no significant changes in emission intensity observed outside strongly acidic conditions (see Figure S9, Supplementary Materials). Among the investigated metal salts, the highest FE values were observed for Cu(II) and Sn(II), exceeding **4**, and to a lesser extent for Pb(II). This behavior indicates a discriminative sensing response arising from the different affinities of the dimethylaminoethyl receptor toward various metal cations.

Such discriminative sensing behavior is typically rationalized in terms of the Hard–Soft Acid–Base (HSAB) concept, as well as coordination geometry and ligand-field effects. Although selectivity was evaluated using individual metal ions, it should be noted that performance in competitive systems may differ due to interference effects. Therefore, additional studies in the presence of multiple metal ions would be required for a comprehensive assessment of selectivity under practical conditions [36,37]. In our case, no fluorescence response was observed toward Ag(I), Co(II), and Mg(II), while only moderate fluorescence enhancement was detected for Zn(II) and Cd(II). Although HSAB considerations can explain the lack of response toward hard metal cations such as Mg(II), Ca(II), and Ba(II), the overall sensing pattern is more accurately described by a combination of coordination geometry and solvent competition effects. Efficient suppression of the PET process requires sufficiently strong and geometrically compatible binding of the metal ion to the dimethylaminoethyl receptor. In this context, the pronounced fluorescence enhancement observed for Cu(II) and Sn(II) is consistent with their favorable HSAB characteristics and flexible coordination geometries, which enable strong interaction with the tertiary amine donor and effective PET suppression even in a coordinating solvent. In contrast, highly charged cations that preferentially bind to oxygen donors and are strongly solvated in DMF, such as Al(III) and Cr(III), are not expected to induce fluorescence enhancement. The stoichiometric ratio of Cu(II) and **3b** in their complex was determined using the method of continuous variations, by plotting (F0 − F)×(1 − X), where F and F0 are the emissions of the probe in the absence and presence of Cu^2+^, respectively, against the molar ratio of Cu^2+^ (X = [Cu^2+^]/([**3b**] + [Cu^2+^])). As shown in Figure S10 in the Supplementary Materials, the Job’s plot analysis confirmed that the complex formed between **3b** and Cu(II) has a 1:1 stoichiometry, which was further used as a basis for the subsequent studies. The binding affinity of **3b** toward Cu(II) was evaluated using the Benesi–Hildebrand double reciprocal method, assuming a 1:1 complex formation as supported by the Job’s plot analysis. The calculated binding constant is on the order of 1 × 10^4^ M^−1^, in good agreement with previously reported naphthalimide-based Cu(II) sensors [38,39]. The limit of detection (LoD) was estimated from the linear region of the fluorescence titration curve (prior to saturation) using the 3σ/k method, where *k* is the slope of the calibration plot, and *σ* is the standard deviation of three independent measurements of the blank solution. The obtained LoD is in the 10^−8^ M range, indicating good sensitivity of the sensor toward Cu(II).

It is worth noting that no significant changes were observed in the absorption spectra of **3b** upon addition of Cu(II). This behavior is consistent with the assignment of the lowest-energy transition as a π–π* excitation with negligible charge-transfer character. As a result, coordination of the metal ion at the imide moiety does not substantially perturb the ground-state electronic structure of the chromophore, while the fluorescence response is governed by suppression of the PET process in the excited state.

To further elucidate the complex formation of the symmetrically benzofuran-annulated NI bearing a dimethylaminoethyl receptor fragment, molecular modeling studies of its 1:1 Cu(II) complexes were performed. Three different coordination modes of **3b** with Cu(II) were investigated. All structures were fully optimized without imposing any symmetry constraints. Thermodynamic parameters (electronic energies, enthalpies, and Gibbs free energies) for complex formation were calculated at the B3LYP/6-31+G(d,p) level of theory according to the reaction:

**3b** + [Cu(H_2_O)_6_]^2+^ → [(**3b**)Cu(H_2_O)n]^2+^ + mH_2_O

where *n* = 3 or 4 and *m* = 3 or 2, respectively, using DMF as solvent. Vibrational frequency analysis was performed at the same level of theory, and no imaginary frequencies were found for the optimized structures, confirming that all geometries correspond to local minima on the potential energy surface. The calculated energy changes for the complexation reactions are summarized in Table 3.

**Figure 8:**
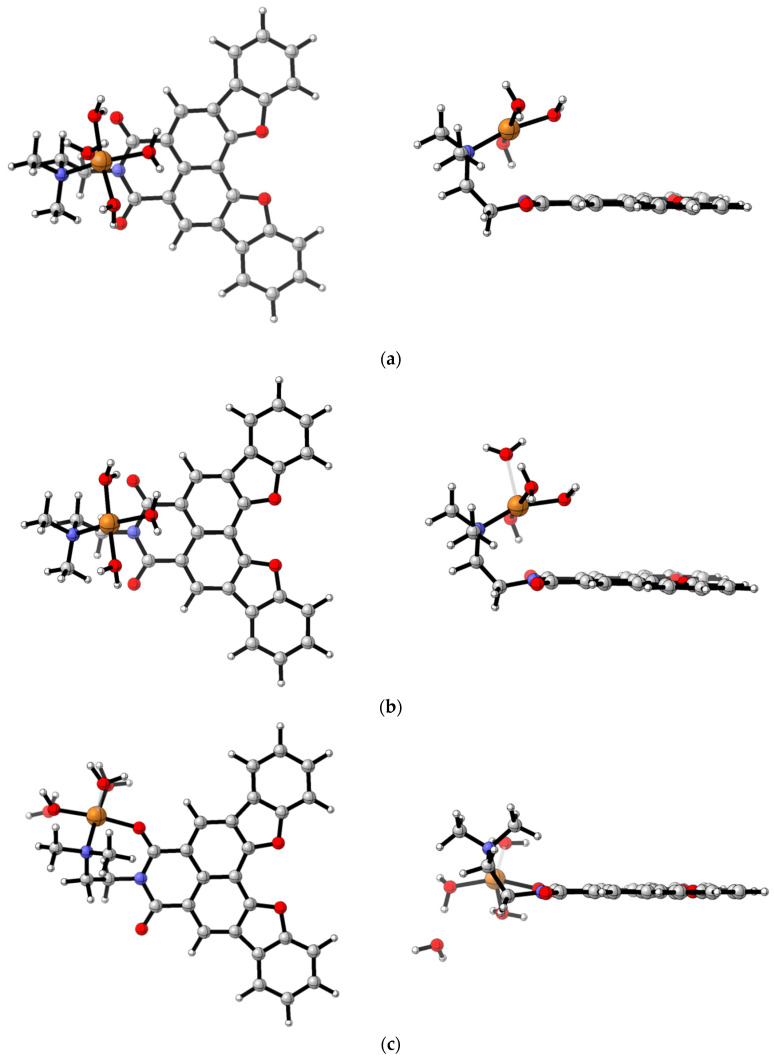
PBE0/6-31+G(d,p)-optimized structures of **3b** complexes with Cu^2+^ ions: (**a**) complex with 3 water molecules; (**b**) complex with 4 water molecules; (**c**) complex with 4 water molecules, different arrangement. Color palette: light gray—carbon atoms, white—hydrogen atoms, red—oxygen atoms, blue—nitrogen atoms, orange—Cu^2+^ ions.

The optimized complexes contain either three or four coordinated water molecules (Figure 8). In the first complex (Figure 8a, entry 1 in Table 3), the Cu(II) center adopts a coordination number of four, whereas in the remaining two complexes (Figure 8b,c) a coordination number of five is observed. In the latter case (Figure 8c), the initial octahedral arrangement of water molecules around the Cu(II) ion was not preserved during geometry optimization. Instead, reorganization of the first solvation shell occurred, with one water molecule displaced into the second coordination sphere. In the third complex (entry 3 Table 3, Figure 8c), Cu(II) coordinates to one of the naphthalimide oxygen atoms, resulting in a more stable structure than the other five-coordinate complex.

In all studied cases, the negative Gibbs free energies of complex formation with hydrated Cu^2+^ ions in DMF (Table 3, last column) indicate a spontaneous and thermodynamically favorable complexation process. These results indicate that the sensing response is governed by coordination-driven equilibrium processes, as commonly observed for PET-based fluorescent sensors [37,40,41]. In solution, such reversible behavior may be further confirmed by chelator-assisted metal ion removal (e.g., EDTA), although this was beyond the scope of the present study.

The optimized structures reveal that the preferred coordination mode involves binding of the metal ion to one nitrogen atom from the dimethylaminoethyl substituent and one oxygen atom from the imide moiety of the dye (Figure 8c, entry 3 in Table 3, ΔG = −34.5 kcal/mol). This chelating interaction effectively engages the lone pair of the tertiary amine, thereby suppressing the PET process. Consistent with this theoretical finding, Cu(II) induces one of the highest fluorescence enhancement (FE) factors observed experimentally, confirming that strong and geometrically compatible metal coordination is a key requirement for efficient PET blocking in this system. To further elucidate the electronic structure of the (**3b**)·Cu(II) complex and its relation to the PET process, the frontier molecular orbitals were analyzed (Figure S13, Supplementary Materials). The HOMO is predominantly localized on the imide fragment, in contrast to unbound **3b** (see Figure 4), which is consistent with a PET-active system. This electronic redistribution provides a clear rationale for the experimentally observed fluorescence enhancement upon complex formation.

In contrast to the benzofuran-annulated system, the dioxin-annulated NI derivative **13b** did not exhibit a sensing response toward the investigated metal cations. This outcome is consistent with the electronic structure of the receptor–chromophore assembly and reflects an unfavorable energetic alignment between the donor and acceptor units. In this case, the nitrogen-centered donor orbital of the receptor is not sufficiently high in energy to enable reductive PET toward the excited NI chromophore, rendering this molecular design ineffective for switch-ON PET sensing. Consequently, no further complexation studies or computational modeling were pursued for this system. Notably, titration with Cu(II) even led to additional fluorescence quenching, most likely arising from paramagnetic effects rather than productive coordination-induced PET suppression.

## 3. Conclusions

In this work, a series of 1,8-naphthalimide derivatives bearing extended heterocyclic systems and, where applicable, a dimethylaminoethyl receptor fragment at the imide nitrogen were synthesized and systematically investigated with respect to their photophysical and optical sensing properties. Benzofuran- and benzodioxin-annulated NIs were obtained using tailored synthetic strategies that enable efficient π-system extension while addressing solubility and purification challenges associated with highly conjugated imide frameworks.

Spectroscopic studies revealed pronounced differences between the two annulation motifs. Benzofuran-annulated NIs display structured absorption bands characteristic of π–π* transitions and strong fluorescence emission, whereas introduction of a dimethylaminoethyl receptor induces efficient fluorescence quenching via a reductive photoinduced electron transfer process. In contrast, benzodioxin-annulated derivatives exhibit broad, structureless absorption bands assigned to intramolecular charge-transfer transitions and inherently low fluorescence quantum yields in polar media, accompanied by large Stokes shifts.

Density functional theory and time-dependent DFT calculations provided insight into the electronic structures of the studied systems and successfully rationalized the experimentally observed photophysical trends. In the benzofuran-annulated series, the energetic alignment of donor and acceptor orbitals is favorable for reductive PET and can be efficiently modulated by analyte binding. Molecular modeling of Cu(II) complexes further demonstrated that strong and geometrically compatible metal coordination involving the receptor nitrogen and the imide oxygen effectively suppresses PET, in agreement with the experimentally observed fluorescence enhancement.

Fluorescence titration experiments confirmed that the receptor-bearing benzofuran-annulated NI functions as a switch-ON PET sensor for protons and selected metal cations, with pronounced responses toward Cu(II) and Sn(II). The observed selectivity arises from a combination of favorable donor–acceptor energetics, coordination geometry, and solvent effects. Overall, the results highlight how controlled heterocyclic annulation within naphthalimide frameworks governs their electronic structure and sensing performance, providing useful guidelines for the rational design of PET-based optical sensors.

## Figures and Tables

**Table 1:** Photophysical characteristics of the studied compounds measured in DMF: lowest-energy absorption maximum ($\lambda_{max}^{ABS}$, nm), molar absorptivity (ε, L mol^−1^ cm^−1^), emission maximum ($\lambda_{max}^{FLU}$, nm), Stokes shift (${\Delta \nu }_{St}$, cm^−1^), and fluorescence quantum yield (QY, %).

Compound	$\lambda_{max}^{ABS}$, nm	ε, L mol^−1^ cm^−1^	$\lambda_{max}^{FLU}$, nm	${\Delta \nu }_{St}$, cm^−1^	QY, %
**3a**	389	12,300	414	1552	26.9
**3b**	387	10,800	414	1685	4.9
**13a**	417	6780	535	5289	5.2
**13b**	417	6713	535	5289	2.1

**Table 2:** Theoretically calculated absorption maxima (in eV), values of oscillator strength (f), and molecular orbital contributions to electronic transition in DMF for **3a,b** and **13a,b** compared with experimental absorption maxima.

	PBE0/6-311+G(2d,p)			Experiment
	Electronic Transition Contribution	λ_max_ [eV]	f	λ_max_ [eV]
**3a**	HOMO → LUMO	3.25	0.2481	3.19
**3b**	HOMO-1 → LUMO	3.26	0.1789	3.2
**13a**	HOMO → LUMO	2.73	0.1209	2.97
**13b**	HOMO → LUMO	2.72	0.1184	2.97

**Table 3:** The change in electron energy, zero-point corrected energy, enthalpy and Gibbs energies (in kcal mol^−1^) for the complex formation reactions of **3b** with hydrated Cu^2+^ cations in DMF solvent (ΔG^37^).

Reaction	ΔE	ΔE_zpc_	ΔH	ΔG
**3b** + [Cu(H_2_O)_6_]^2+^ → [(**3b**)Cu(H_2_O)_3_]^2+^ + 3H_2_O	−21.3	−24.0	−23.6	−33.6
**3b** + [Cu(H_2_O)_6_]^2+^ → [(**3b**)Cu(H_2_O)_4_]^2+^ + 2H_2_O	−21.7	−22.2	−22.2	−23.1
**3b** + [Cu(H_2_O)_6_]^2+^ → [(**3b**)Cu(H_2_O)_4_]^2+^ + 2H_2_O	−29.2	−31.3	−30.4	−34.5

## Data Availability

The original contributions presented in this study are included in the article/Supplementary Material. Further inquiries can be directed to the corresponding author.

## Supplementary Materials

The following supporting information can be downloaded at https://www.mdpi.com/article/10.3390/s26072236/s1: Materials and Methods. Figure S1. 1H NMR spectrum of **3b** in trifluoroacetic acid-d. Figure S2. 13C NMR spectrum of **3b** in trifluoroacetic acid-d. Figure S3. 1H NMR spectrum of **13a** in chloroform-d. Figure S4. 13C NMR spectrum of **13a** in chloroform-d. Figure S5. FT-IR spectrum of **3a**. Figure S6. FT-IR spectrum of **3b**. Figure S7. FT-IR spectrum of **13a**. Figure S8. FT-IR spectrum of **13b**. Figure S9. Influence of pH on the fluorescence intensity of **3b**. Figure S10. Job’s plot analysis of the **3b** + Cu(II) complex formation. Figure S11. Determination of **3b** detection limit towards Cu(II). Figure S12. Determination of the Cu(II) binding constant K (Benesi–Hildebrand double reciprocal plot for 1:1 stoichiometry) of **3b**. Figure S13. Shape representation of HOMO and LUMO of **3b** complex with Cu^2+^ ions in DMF from PBE0/6-31+G(d,p) computations.

## Abbreviations

The following abbreviations are used in this manuscript:

PET	Photoinduced Electron Transfer
NI	1,8-Naphthalimide
DFT	Density Functional Theory
TDDFT	Time-dependent DFT
NMP	1-Methylpyrrolidin-2-one (N-Methyl-2-Pyrrolidone)
DMF	*N*,*N*-Dimethylformamide
TLC	Thin-Layer Chromatography
FQ	Fluorescence Quenching
FE	Fluorescence Enhancement
ICT	Intramolecular Charge Transfer
