# Supplementary material for: Naphthalimide Derivatives with Extended Heterocyclic Systems—Synthesis, Spectral and Sensing Properties
Source: Sensors (Basel). 2026 Apr 4;26(7):2236. doi: 10.3390/s26072236 (PMC13075197; doi:10.3390/s26072236)
Supplement: Supplementary file 1 [file sensors-26-02236-s001.zip › sensors-4207415-supplementary.pdf]

Naphthalimide derivatives with extended heterocyclic systems – synthesis, spectral and sensing properties

Hristo Manov [1], Ivo Grabchev *[2], Yulian Zagranyarski [1], Diana Cheshmedzhieva [1], Ivan Atanasov [1], Monika Mutovska [1], Konstantin Konstantinov [3], and Stanimir Stoyanov [1*]

[1] Faculty of Chemistry and Pharmacy, Sofia University "St. Kliment Ohridski", 1 J. Baurchier Blvd., 1164 Sofia, Bulgaria.
[2] Faculty of Medicine, Sofia University "St. Kliment Ohridski", 1 Koziak Str., 1407 Sofia, Bulgaria.
[3] Faculty of Pharmacy, Medical University of Sofia, 2 Dunav Str., 1000 Sofia, Bulgaria
* Correspondence: sstoyanov@chem.uni-sofia.bg (S.S); i.grabchev@chem.uni-sofia.bg (I.G.).

Supplementary materials

Table of Figures:

Figure S1. ^{1}H NMR spectrum of **3b** in trifluoroacetic acid-d. 3
Figure S2. ^{13}C NMR spectrum of **3b** in trifluoroacetic acid-d. 3
Figure S3. ^{1}H NMR spectrum of **13a** in chloroform-d....4
Figure S4. ^{13}C NMR spectrum of **13a** in chloroform-d. 4
Figure S5. FT-IR spectrum of **3a**. 5
Figure S6. FT-IR spectrum of **3b**. 5
Figure S7. FT-IR spectrum of **13a**. 6
Figure S8. FT-IR spectrum of **13b**. 6
Figure S9. Influence of pH on the fluorescence intensity of **3b**. 7
Figure S10. Job's plot analysis of the **3b**+Cu(II) complex formation. 7
Figure S11. Determination of **3b** detection limit towards Cu(II)....8
Figure S12. Determination of the Cu(II) binding constant K (Benesi-Hildebrand double reciprocal plot for 1:1 stoichiometry) of **3b**. 8
Figure S13. Shape representation of HOMO and LUMO of **3b** complex with Cu^{2+} ions in DMF from PBE0/6-31+G(d,p) computations. 9

1. Materials and Methods

1.1. Synthesis

7-(2-(dimethylamino)ethyl)-6H-benzo[2,3]benzofuro[5,6,7-de]benzo[4,5]furo[2,3-g]isoquinoline-6,8(7H)-dione (**3b**)

To a suspension of the anhydride **6** (2 mmol) in 20 mL of 2-methyl-2-butanol was added *N*^{1},*N*^{1}-dimethylethane-1,2-diamine (3 mmol, 0.26 g). The reaction mixture was refluxed for 90 min. After cooling, about 20 g of crushed ice was added to the reaction mixture, and the resulting crystals were filtered, washed abundantly with water, and dried. The resulting imide was of high purity and was used without further purification. Yield 0.85 g (95 %).

^{1}H NMR (500 MHz, trifluoroacetic acid-d) δ 8.88 (d, J = 1.3 Hz, 2H), 7.93 (d, J = 7.6 Hz, 2H), 7.59-7.56 (m, 4H), 7.47 (ddd, J = 7.9, 4.8, 3.1 Hz, 2H), 4.48 (t, J = 5.5 Hz, 2H), 3.65-3.58 (m, 2H), 3.15 (s, 6H). ^{13}C NMR (126 MHz, trifluoroacetic acid-d) δ 168.77, 159.46, 156.24, 131.27, 128.80, 128.76, 127.14, 124.84, 124.17, 122.88, 117.24, 114.36, 108.17, 60.29, 46.16, 38.46. FTIR (cm^{-1}): 2993, 2855, 2775, 1689, 1654, 1638, 1583, 1389, 1340, 1188, 1043, 745. Anal. calcd. $C_{28}H_{20}N_2O_4$ C, 74.99; H, 4.50; N, 6.25 %; found C, 74.92; H, 4.27; N, 6.13 %.

5-(2-ethylhexyl)-4H-benzo[de]benzo[5,6][1,4]dioxino[2,3-g]isoquinoline-4,6(5H)-dione (**13a**)

A mixture of 5,6-dibromo-2-(2-ethylhexyl)-1*H*-benzo[*de*]isoquinoline-1,3(2*H*)-dione **12** (5.0 mmol, 2.34 g), catechol (6.0 mmol, 0.66 g) and potassium carbonate (12 mmol, 1.66 g) in 25 mL NMP was stirred and heated at 150 °C for 50 min under argon. The mixture was cooled down to room temperature and poured into 50 mL of cold water containing 5 mL of concentrated hydrochloric acid. The precipitation was filtered, washed with water, and dried. The crude product was purified by column chromatography on silica gel using cyclohexane/dichloromethane as the eluent; the chromatographic system was started with pure cyclohexane, and the polarity gradually increased by addition of dichloromethane up to 30 %. Yield 1.91 g (92 %).

^{1}H NMR (500 MHz, Chloroform-d): 8.45 (dd, J = 7.2, 1.1 Hz, 1H), 8.31 (dd, J = 8.4, 1.1 Hz, 1H), 8.05 (s, 1H), 7.68 (dd, J = 8.4, 7.3 Hz, 1H), 7.00 – 6.96(m, 3H), 6.91 – 6.89 (m, 1H), 4.06 (qd, J = 12.9, 7.3 Hz, 2H), 1.91 (hept, J = 6.2 Hz, 1H), 1.42 – 1.25 (m, 8H), 0.92 (t, J = 7.4 Hz, 3H), 0.88 (t, J = 7.1 Hz, 3H). ^{13}C{^{1}H} NMR (Chloroform-d, 126 MHz): 164.49, 163.47, 141.80, 141.32, 141.25, 139.17, 130.51, 127.22, 126.66, 125.91, 125.44, 124.74, 122.72, 122.23, 121.77, 118.17, 116.94, 116.80, 44.30, 38.05, 30.87, 28.84, 24.18, 23.23, 14.24, 10.78.

FTIR (cm^{-1}): 3071, 2958, 2852, 1696, 1659, 1589, 1494, 1368, 1253, 1205, 780, 744. Anal. calcd. $C_{26}H_{25}NO_4$ C, 75.16; H, 6.07; N, 3.37 %; found C, 74.99; H, 5.95; N, 3.43 %.

NMR spectra were recorded on a Bruker Avance 500 MHz instrument (Bruker, Karlsruhe, Germany) operating at 500 and 126 MHz for ^{1}H and ^{13}C, respectively. Chloroform-d and trifluoroacetic acid-d were used as solvents. Chemical shifts are reported in δ units (ppm) and referenced to the residual solvent signals (^{1}H at 7.26 ppm and ^{13}C at 77.160 ppm for chloroform-d and ^{1}H at 11.50 ppm and ^{13}C at 164.20 ppm for trifluoroacetic acid-d). Elemental analyses were carried out on a Leco CHNS-932 (Leco Europe, Geleen, The Netherlands). Thin layer chromatographic (TLC) analysis was performed on silica gel plates (Macherey-Nagel F60 254 40 × 80; 0.2 mm, Macherey-Nagel, Duren, Germany).

2.2. Spectroscopic measurements

Absorption and emission spectra were recorded on Varian Cary 5000 UV/Vis/NIR and Cary Eclipse spectrometers (Varian, Mulgrave, Australia), respectively. Absolute Fluorescence Quantum Yields were measured on a PerkinElmer FL 8500 spectrometer (PerkinElmer, Shelton, USA) with an integrating sphere, using DeMello method, correcting for the indirect absorption/emission. Spectroscopy grade *N,N*-dimethylformamide (DMF) (Fisher Scientific, Hampton, NH, USA) was used as a solvent. All metal salts used in the optical studies were anhydrous nitrates.

2.3. Computational details

The geometry optimization and photophysical properties of compounds were modeled with G16 software package. The optimization of the ground and excited state geometry for **3a,b** and **13a**,**b** was performed within DFT and TDDFT formalisms, respectively. The theoretical computations have been carried out using PBE0/6-311+G(2d,p) levels of theory. Vibrational frequencies were evaluated for each structure at the same method/basis set to verify that the structures are indeed a minimum of the potential energy surface, and no imaginary frequency was found.

The absorption wavelengths were determined by TDDFT calculations of vertical excitations. The absorption spectra of the compounds were simulated by TDDFT using the same functional and basis set- PBE0/6-311+G(2d,p). Six or twelve excited singlet states were studied. The lowest energy transition with non-zero oscillator strength is considered for each molecule. To simulate fluorescence, the optimization of the excited state, corresponding to transition of interest was performed at TDDFT, starting from the ground state geometry. Calculations of the vibrational frequencies and the absence of imaginary frequencies confirm that the excited state geometries are minimum on the potential energy surface. The fluorescence electronic transitions were calculated as vertical de-excitations based on the optimized geometries of the excited state. A detailed description of the procedure can be found elsewhere. Solvent effects were examined at each step by means of PCM formalism. All the computations were performed in DMF as a solvent. Cu^{2+} complexes of **3b** have been optimized at B3LYP/6-31+g(d,p) level of theory in DMF.

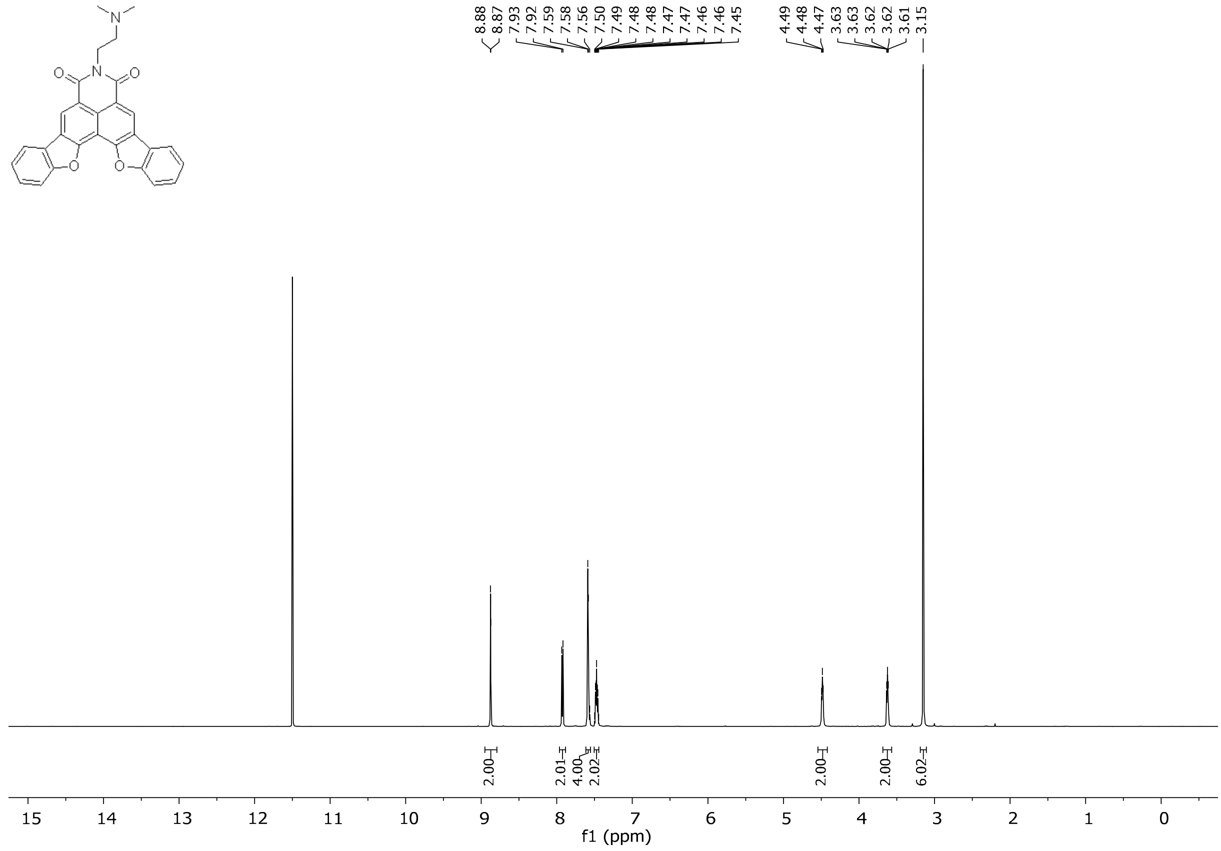

*Figure S1. ^{1}H NMR spectrum of **3b** in trifluoroacetic acid-d.*

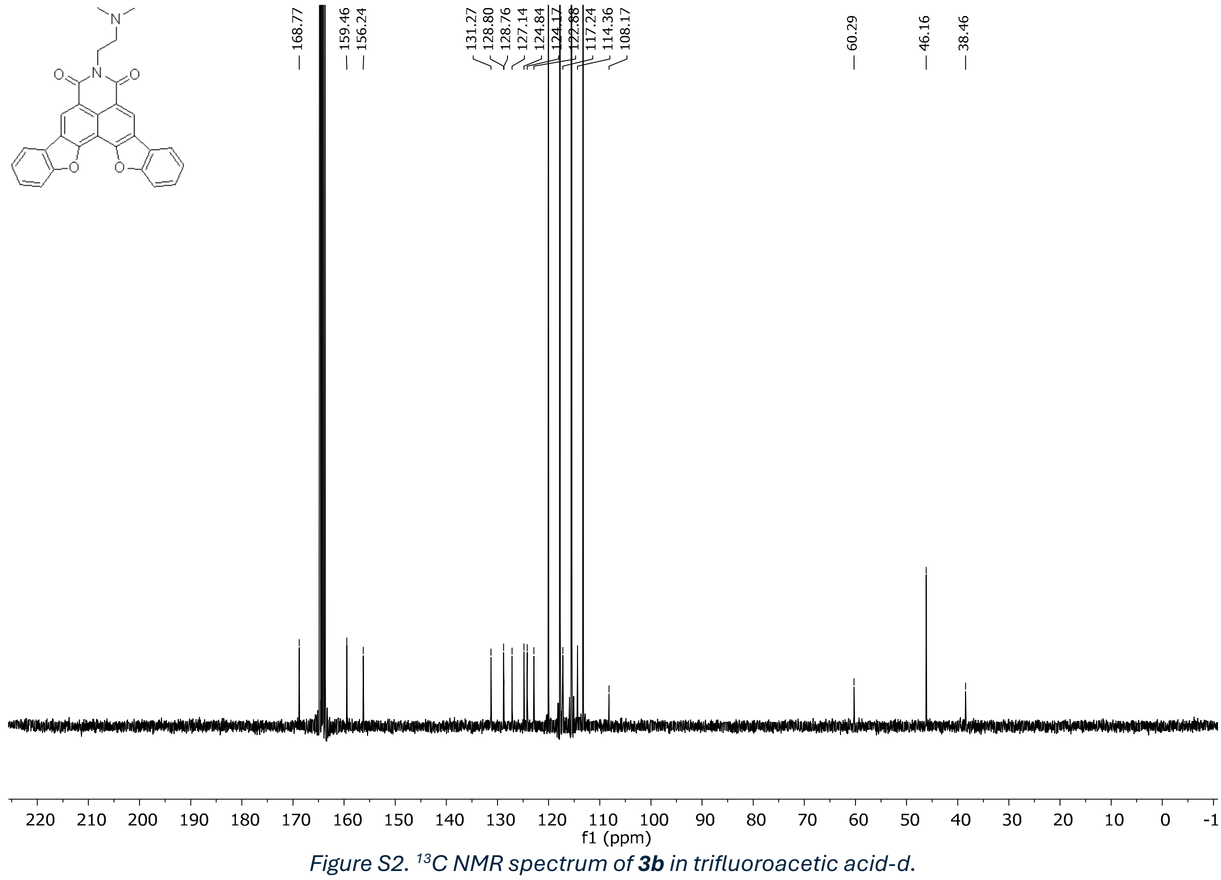

*Figure S2. ^{13}C NMR spectrum of **3b** in trifluoroacetic acid-d.*

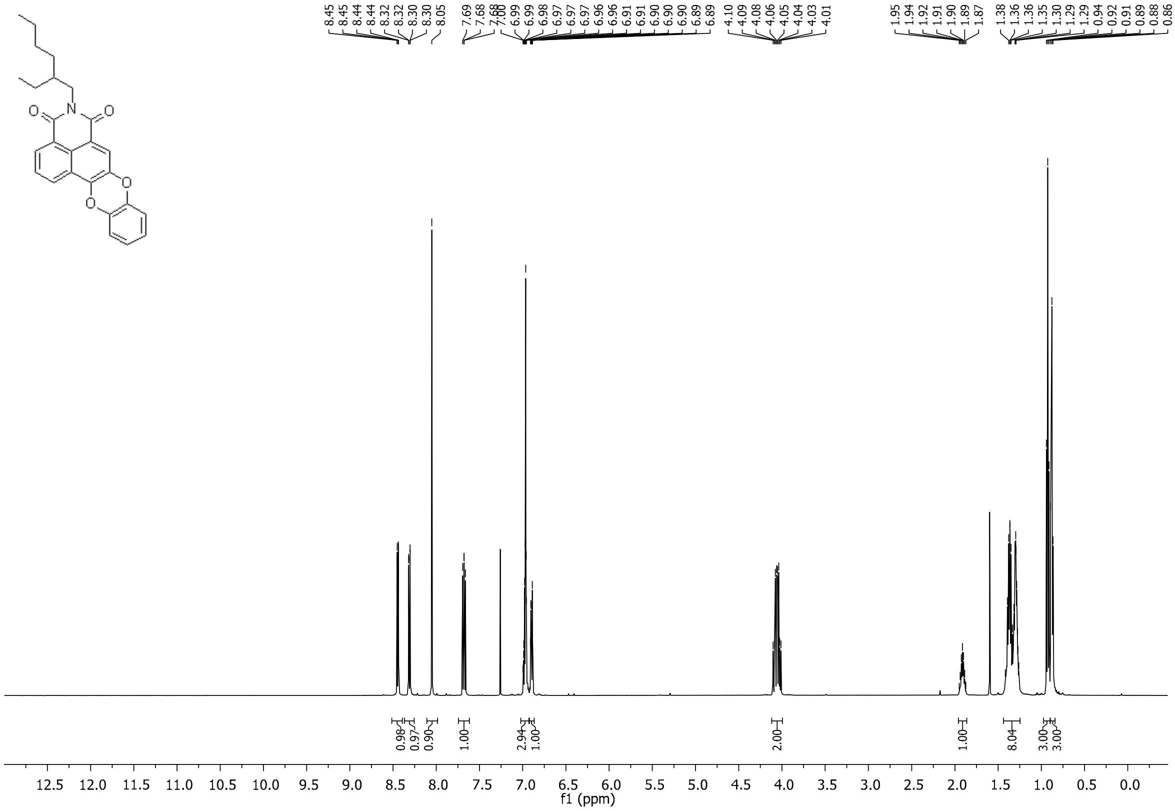

*Figure S3. ^{1}H NMR spectrum of **13a** in chloroform-d.*

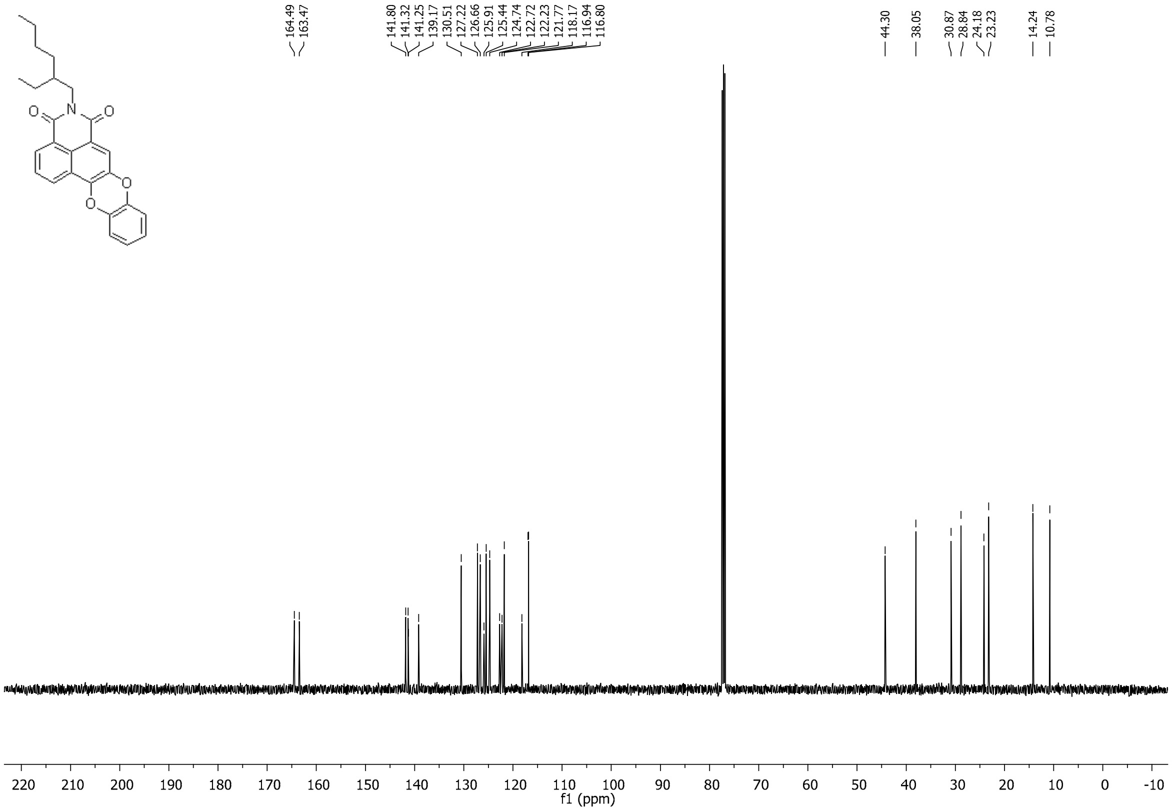

*Figure S4. ^{13}C NMR spectrum of **13a** in chloroform-d.*

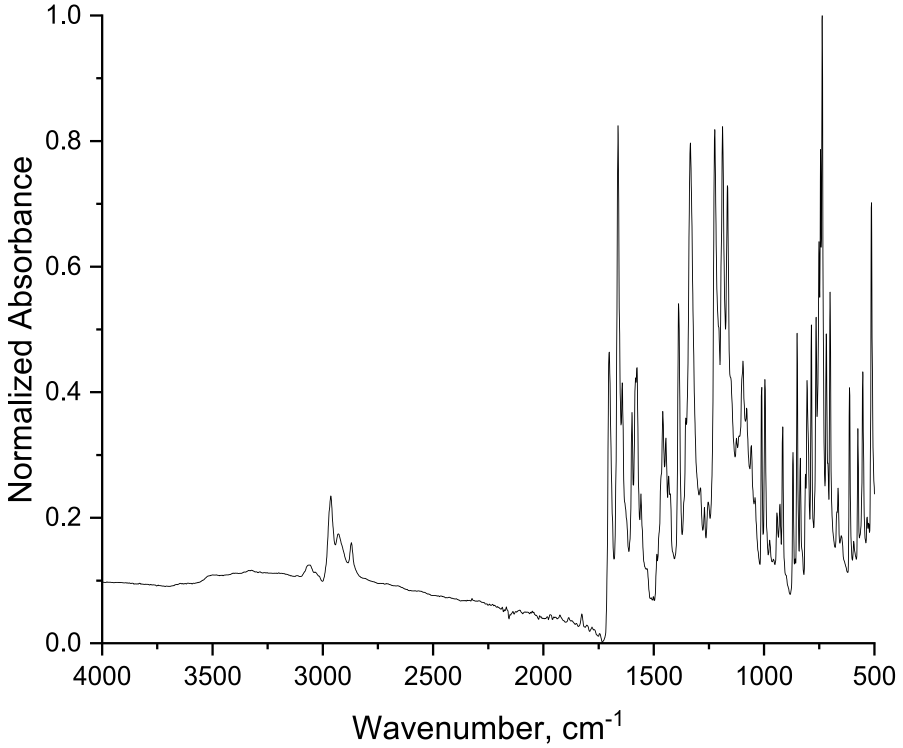

FTIR (cm^{-1}): 2963, 2866, 1700, 1662, 1577, 1385, 1333, 1223, 1183, 736.

Figure S5. FT-IR spectrum of ***3a****.*

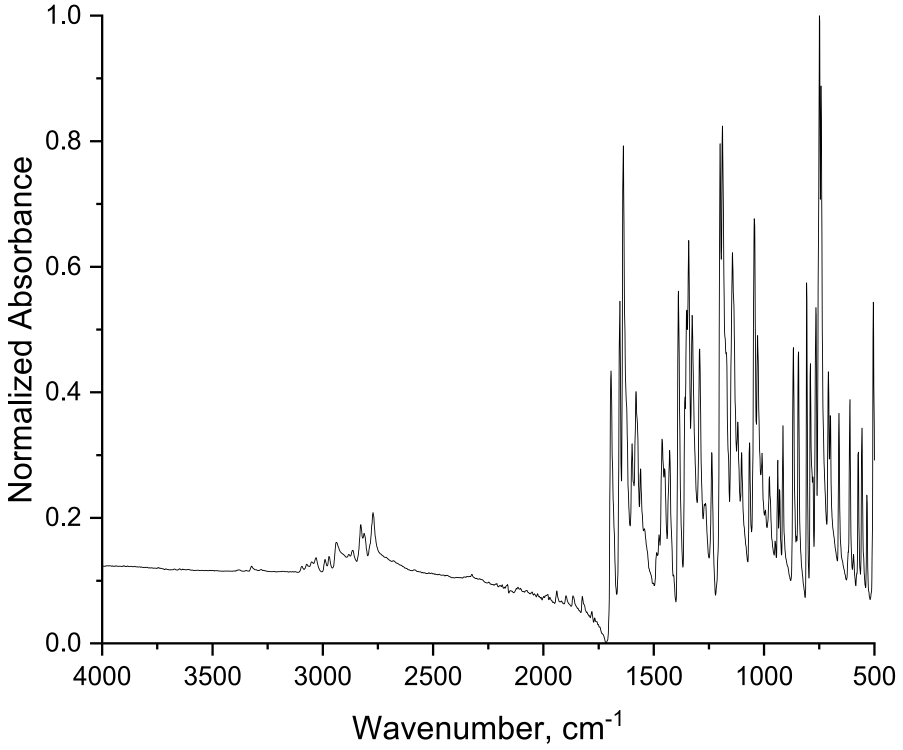

FTIR (cm^{-1}): 2993, 2855, 2775, 1689, 1654, 1638, 1583, 1389, 1340, 1188, 1043, 745.

Figure S6. FT-IR spectrum of ***3b****.*

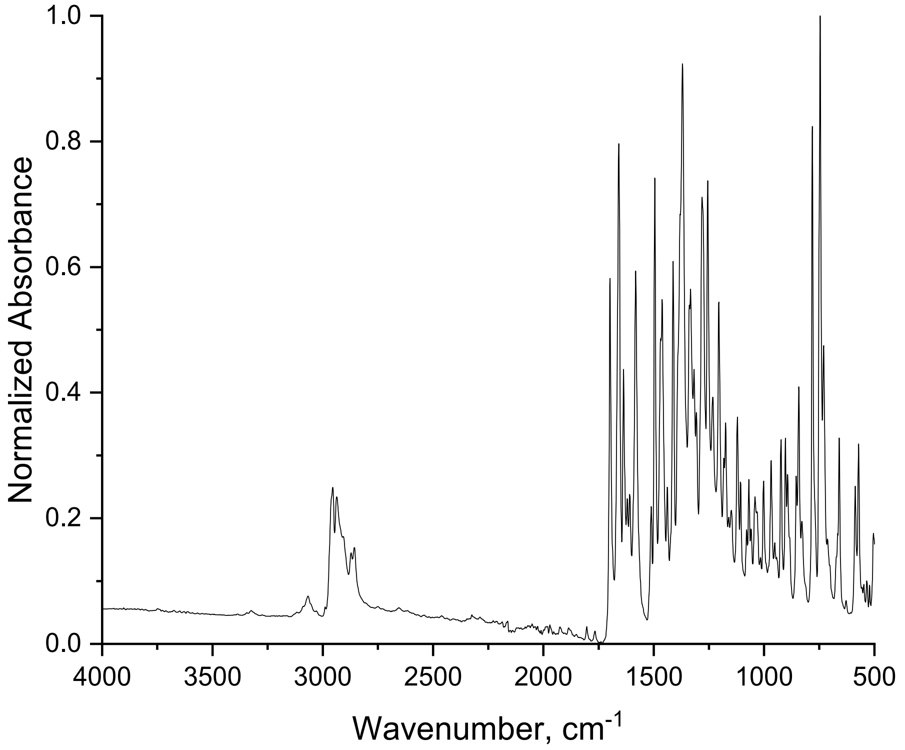

FTIR (cm^{-1}): 3071, 2958, 2852, 1696, 1659, 1589, 1494, 1368, 1253, 1205, 780, 744.

*Figure S7. FT-IR spectrum of **13a**.*

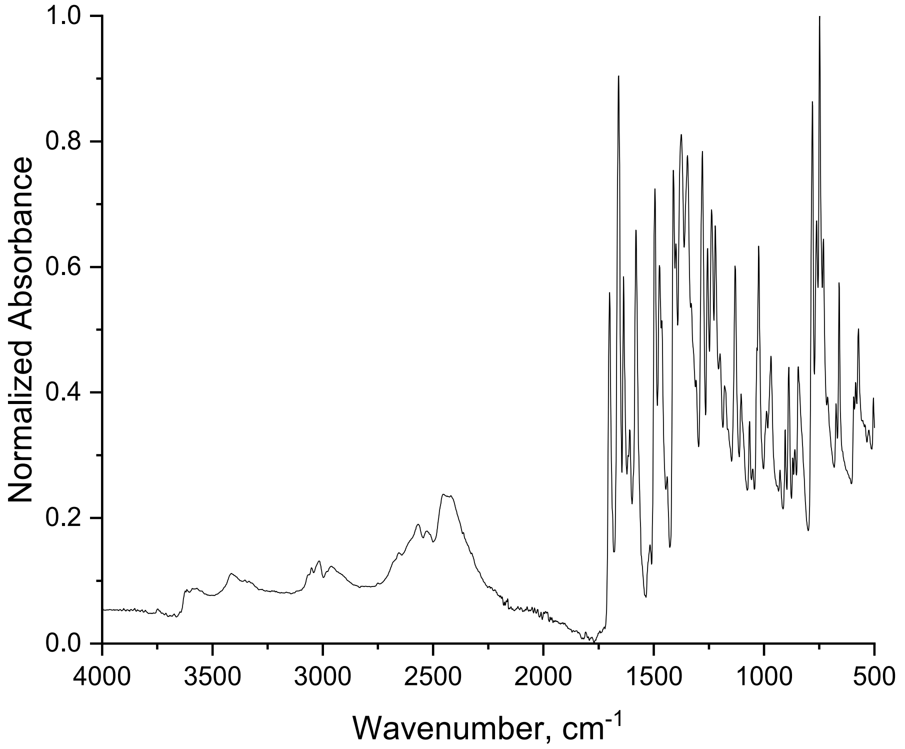

FTIR (cm^{-1}): 3008, 2958, 1699, 1659, 1637, 1579, 1494, 1375, 1280, 1130, 1023, 780, 746.

*Figure S8. FT-IR spectrum of **13b**.*

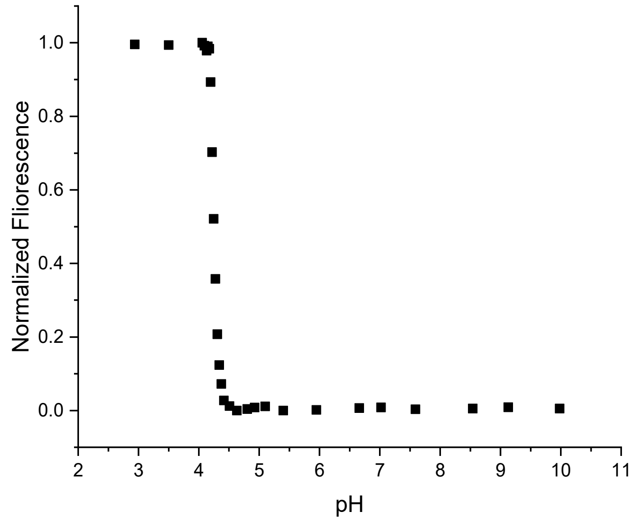

*Figure S9. Influence of pH on the fluorescence intensity of **3b**.*

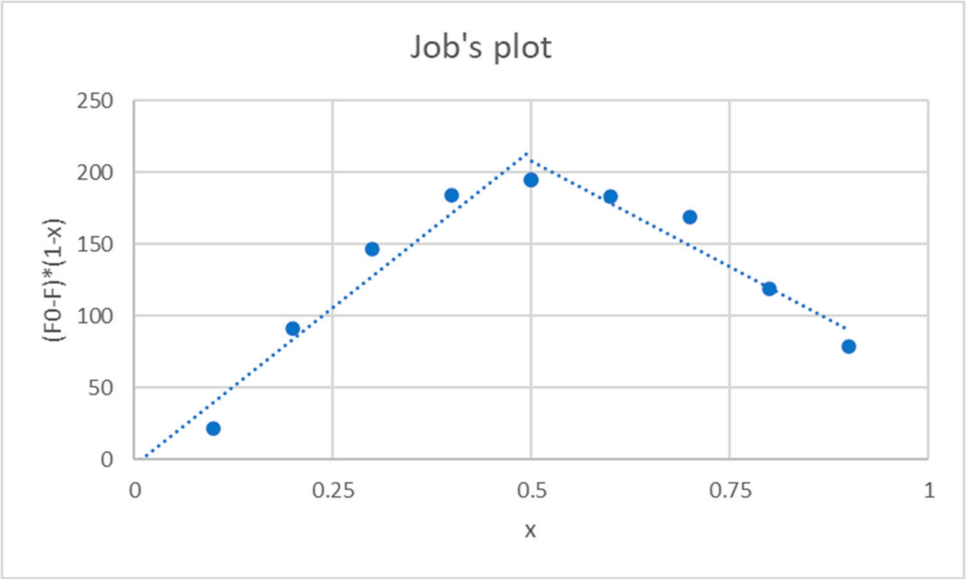

*Figure S10. Job's plot analysis of the **3b**+Cu(II) complex formation.*

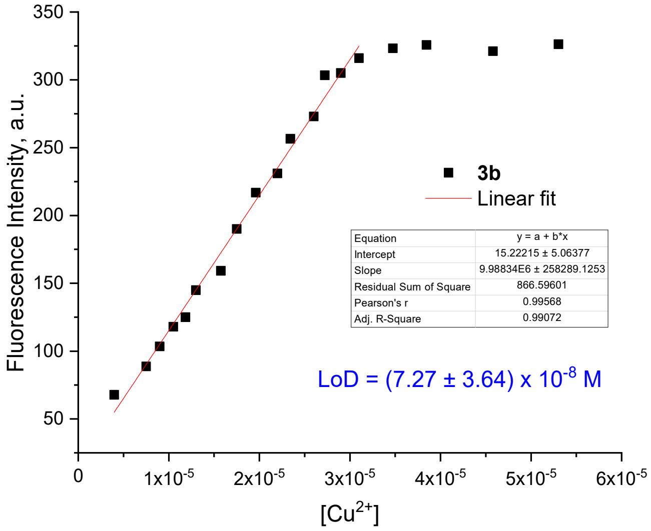

Figure S11. Determination of ***3b*** *detection limit towards Cu(II).*

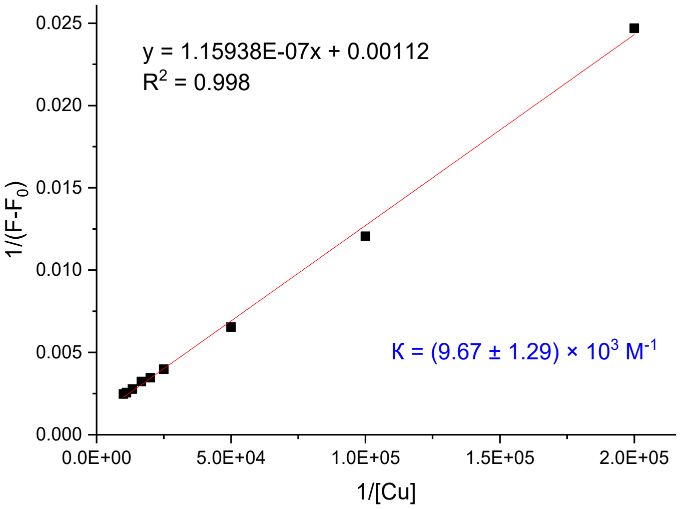

Figure S12. Determination of the Cu(II) binding constant K (Benesi-Hildebrand double reciprocal plot for 1:1 stoichiometry) of ***3b****.*

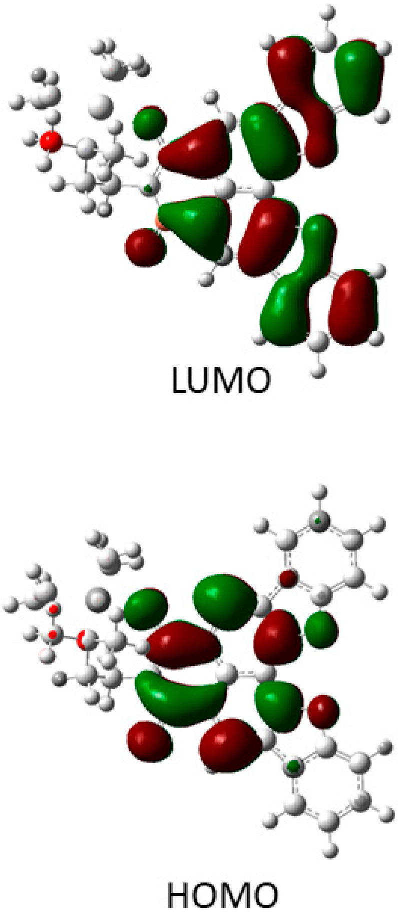

Figure S13. Shape representation of HOMO and LUMO of **3b** complex with Cu^{2+} ions in DMF from PBE0/6-31+G(d,p) computations.
